# Sex and limb impact biomechanics associated with risk of injury during drop landing with body borne load

**DOI:** 10.1371/journal.pone.0211129

**Published:** 2019-02-06

**Authors:** Kayla D. Seymore, AuraLea C. Fain, Nicholas J. Lobb, Tyler N. Brown

**Affiliations:** Center for Orthopaedic & Biomechanics Research, Boise State University, Boise, ID, United States of America; Nanyang Technological University, SINGAPORE

## Abstract

Increasing lower limb flexion may reduce risk of musculoskeletal injury for military personnel during landing. This study compared lower limb biomechanics between sexes and limbs when using normal and greater lower limb flexion to land with body borne load. Thirty-three participants (21 male, 12 female, age: 21.6±2.5 years, height: 1.7±0.1 m, weight: 74.5±9.0 kg) performed normal and flexed lower limb landings with four body borne loads: 20, 25, 30 and 35 kg. Hip and knee biomechanics, peak vertical ground reaction force (GRF), and the magnitude and direction of the GRF vector in frontal plane were submitted to two separate repeated measures ANOVAs to test the main and interaction effects of sex, load, and landing, as well as limb, load, and landing. Participants increased GRFs (between 5 and 10%) and hip and knee flexion moments when landing with body borne load, but decreased vertical GRF 19% and hip adduction and knee abduction joint range of motion and moments during the flexed landings. Both females and the non-dominant limb presented greater risk of musculoskeletal injury during landing. Females exhibited larger GRFs, increased hip adduction range of motion, and greater knee abduction moments compared to males. Whereas, the non-dominant limb increased knee abduction moments and exhibited a more laterally-directed frontal plane GRF vector compared to the dominant limb during the loaded landings. Yet, increasing lower limb flexion during landing does not appear to produce similar reductions in lower limb biomechanics related to injury risk for both females and the non-dominant limb during landing.

## Introduction

Lower limb musculoskeletal injury is a serious health concern for military personnel [1]. According to the U.S. Army, an estimated 75% of recruits will sustain a musculoskeletal injury during basic and/or advanced training [2]. These training-related injuries result in long-term disability and attrition, with substantial financial cost to the Armed Services [1,3]. The most common location of these musculoskeletal injuries is reportedly the knee joint [4]. Recruits frequently suffer bony disorders and sprain, strain, or rupture of the knee’s soft-tissue during military training. These injuries often occur when the joint is forced into dynamic valgus [5,6] during the cutting and landing maneuvers common to military training [7]. Dynamic valgus, described as excessive hip adduction, knee abduction, and ankle eversion joint motions and loads [8,9], may be potentially hazardous for military personnel because of the heavy body borne load they are required to don during training activities. These heavy body borne loads, which routinely range from 20 kg to 45 kg during training-related activities [10], reportedly alter a soldier’s hip and knee biomechanics [11–13], increasing their risk of suffering a musculoskeletal injury [14]—particularly at the knee.

During drop landings between 30 and 100 cm, peak vertical ground reaction force (vGRF) is reportedly between two and five times body weight [15] and significantly increases with the addition of body borne load [11,12]. Soldiers increase their peak vertical GRF between 9% and 17% during a drop landing with heavy military-relevant body borne loads [11]. These elevated landing forces strain the soft-tissues surrounding a joint [8]. As the knee joint and associated musculature are the major contributors to energy absorption during landing [11,15], the joint has potential for musculoskeletal injury from elevated landing forces [16]. Landing forces placed on the knee’s soft-tissues may further increase when the soldier lands with a laterally-directed GRF vector. A laterally-directed GRF vector would act to push the knee into valgus, increasing both knee abduction joint angle and moment–biomechanics implicated in injury at the joint [8,17]. Creaby and Dixon [18] reported the direction of the frontal plane GRF vector is significantly larger for soldiers with a history of lower limb musculoskeletal injury than healthy controls. Considering knee joint loading is reported to increase with the addition of body borne load during military-related activities [13,19], it is warranted to determine whether the lateral direction of GRF vector and/or measures of knee valgus increase when landing with these loads.

Female military personnel are twice as likely to sustain a training-related musculoskeletal injury compared to their male counterparts [4]. This increased injury risk may be attributed to a sex dimorphism exhibited in lower limb biomechanics [20,21], particularly during landing [22,23]. During landing, females’ peak vertical GRF normalized to body weight is reportedly almost two times the magnitude exhibited by males [24]. To attenuate these forces, females use greater hip adduction [21], greater knee valgus (both knee abduction motions and loads), and less hip and knee flexion compared to males when landing without body borne load [25]. During landing, females exhibit greater asymmetries in lower limb biomechanics, including differences in knee valgus between the dominant and non-dominant limb [25,26]. This limb asymmetry may elevate females’ injury risk, as they reportedly suffer more injuries in the non-dominant limb [27,28]. However, it is currently unknown if a similar sex and limb dimorphism in lower limb biomechanics exists when landing with heavy body borne loads.

Increased hip and knee flexion during landing may decrease knee injury risk by promoting greater absorption of the landing forces placed on the musculoskeletal system, both with [11] and without body borne load [29]. During unloaded landings, individuals are able to reduce peak vertical GRF when instructed to increase lower limb flexion [30,31]. Soldiers, however, are reported to decrease hip and knee flexion when landing with heavy military body borne loads [11]. Currently, it is unclear if using greater lower limb flexion when landing with heavy body borne loads is, indeed, attainable for soldiers, and whether it reduces their risk of musculoskeletal injury by decreasing landing forces and knee biomechanics, such as dynamic valgus, related to injury. As such, the purpose of this study was two-fold: (1) to determine whether lower limb biomechanics differed between sexes when using normal and greater lower limb flexion during a drop landing with body borne load, and (2) to determine whether lower limb biomechanics differed between limbs during the normal and flexed landings with load. We hypothesized that females and the non-dominant limb would increase the frontal plane direction of the GRF vector, hip adduction and knee abduction joint angles and moments, and decrease hip and knee flexion joint angles compared to males and the dominant limb. We also hypothesized that these biomechanical parameters would increase with each addition of body borne load, but decrease for both sexes and limbs when using greater lower limb flexion during landing.

## Materials and methods

### Participants

We recruited thirty-three (21 male and 12 female) healthy adults between the ages of 18–40 years (Table 1). To be included, potential participants had to be the proper age, in good health and confirm that they could safely carry loads up to 75 pounds. Potential participants were excluded if they reported current pain or recent injury to the back or lower extremity (previous 6 months), history of back or lower extremity injury or surgery, any known neurological disorder, and/or were currently pregnant. All participants provided written informed consent prior to testing. Research approval was obtained from the Boise State University Institutional Review Board. Approved IRB Protocol Number: 103-MED15-008.

**Table 1:** Demographics for males and females.

	Male (n = 21)	Female (n = 12)	*p*—value
**Age (years)**	21.3 (2.4)	21.9 (2.6)	0.53
**Height (m)**	1.8 (0.1)	1.7 (0.1)	< 0.001
**Mass (kg)**	81.7 (9.7)	67.4 (8.3)	< 0.001

### Protocol

**Fig 1:**
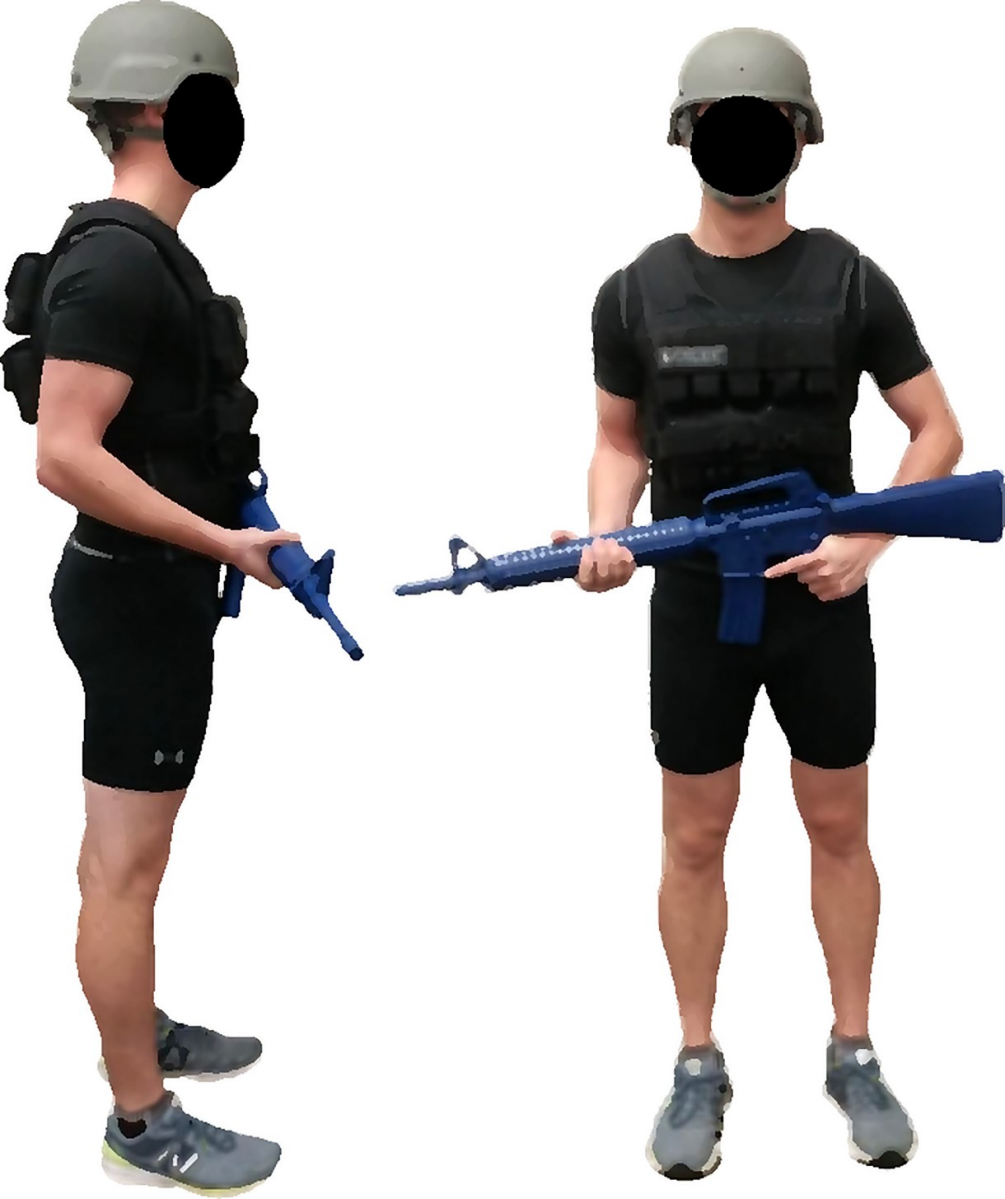
Body borne load configuration.

A repeated-measures research design was used to investigate the impact four military-relevant body borne loads had on lower limb biomechanics exhibited by both sexes (males and females) and limbs (dominant and non-dominant) during a 30 cm drop landing. Participants performed four test sessions. For each session, participants donned a different body borne load (20, 25, 30, and 35 kg). For each load condition, participants were required to wear a standard-issue ACH helmet and carry a mock M16 weapon, with a total mass of approximately 6 kg. Additionally, participants wore a weighted vest (Box, WeightVest.com, Inc. Rexburg, ID, USA), which was systematically adjusted to add the required load (within ± 2%) for each condition to the participant (Fig 1). The testing sequence of each load condition was randomly assign to each participant prior to testing using a 4 x 4 Latin square. Each test session was separated by a minimum of 24 hours to reduce the effects of fatigue and limit chances of injury.

During testing, participants had three-dimensional (3D) lower limb biomechanics recorded during a series of drop landings. During each drop landing, eight high-speed (240 fps) optical cameras (MXF20, Vicon Motion Systems, Ltd., London, UK) captured lower limb motion data and two force platforms (AMTI OR6 Series, Advanced Mechanical Technology, Inc., Watertown, MA) recorded synchronous GRF data (2400 Hz). Prior to testing, all participants completed a footedness questionnaire [32] to establish limb dominance and familiarized themselves with the drop landing maneuver. Participants performed two types of drop landing tasks: normal (NL) and flexed (FL). For the NL, participants were instructed to step off a 30 cm box, land with each foot on a separate force platform, and remain standing for approximately 2 seconds after regaining their balance. Following the NL task, participants performed the FL landings. For the FL, participants were instructed to step off a 30 cm box and exaggerate lower limb flexion upon landing with each foot on a separate force platform. Participants performed five successful trials for each landing task, however, only three successful trials for each condition were submitted to analysis. A drop landing was considered successful if both feet contacted only their assigned force platform during landing. During testing, participants were provided rest between trials to reduce the effects of fatigue.

### Biomechanical analysis

Lower limb joint biomechanics were quantified from the 3D trajectories of 32 retro-reflective markers. After marker placement, each participant stood in anatomical position for a stationary recording used to create a kinematic model in Visual 3D (v6.00, C-Motion, Inc, Germantown, MD, USA). The kinematic model consisted of seven rigid kinematic segments (bilateral foot, shank, and thigh, and pelvis). The pelvis was defined with respect to the global (laboratory) coordinate system and assigned six (three rotation and three translational) degrees of freedom. Functional hip joint centers were calculated according to Rozumalski and Schwartz [33] and local coordinate system assigned three degrees of freedom. Knee and ankle joint centers and local (three degrees of freedom) coordinate systems were calculated in accordance with previous literature using Visual 3D [34,35]. For each drop landing, synchronous GRF and 3D marker trajectories were low-pass filtered with a fourth-order Butterworth filter (12 Hz). The filtered marker trajectories were then processed using Visual 3D to calculate hip and knee joint rotations and expressed relative to each participant’s anatomical position (stationary recording). Joint range of motion (ROM) was calculated as the peak angle minus the initial contact angle.

Filtered kinematic and GRF data were processed using conventional inverse dynamic analysis to obtain resultant 3D joint moments [36]. Inertial properties of each segment were defined according to Dempster et al [37]. Hip and knee resultant moments were defined as flexion-extension, abduction-adduction, and internal-external rotation. The magnitude and direction of the GRF vector in frontal plane were computed according to Creaby and Dixon [18]. The magnitude (GRF_mag_) and direction (GRF_ang_) of the GRF vector in the frontal plane was computed using the following standard trigonometry equations:

$${GRF}_{mag}=\sqrt{F_{x}^{2}+F_{z}^{2}}$$ (1)

$${GRF}_{ang}={tan}^{-1}\frac{F_{x}}{F_{z}}$$ (2)

where F_z_ and F_x_ represent the vertical and mediolateral GRF vectors, respectively. Positive angle in the frontal plane indicated a laterally directed GRF. Joint moments were normalized by body mass (kg) multiplied by height (m) and expressed as external moments, while GRF was normalized to body weight (BW). All biomechanical data were time-normalized to landing phase (initial contact to stabilization) and resampled at 1% increments (N = 101). Initial contact occurred at the first instant GRF exceeded 10 N and stabilization occurred when the combined vGRF of both force plates equaled BW plus the body borne load (N).

### Statistical analysis

The dependent kinematic variables included hip and knee flexion, hip adduction, and knee abduction range of motion (ROM). The dependent kinetic variables included peak of landing phase (0% - 100%) vGRF, GRF_mag_, GRF_ang_, and hip and knee flexion, hip adduction, and knee abduction joint moments. Intraclass correlation coefficients (ICCs) of our primary outcome measures (peak vGRF and hip and knee flexion angles) revealed good to excellent trial-to-trial reliability (range: 0.88–0.97), and thus, subject-based means were quantified for each dependent measure. To test purpose 1, the subject-based mean of each dependent variable was submitted to a 3-way RM ANOVA to test the main effects of and interaction between sex (male, female), load (20, 25, 30, and 35kg), and landing type (NL and FL). To test purpose 2, the subject-based mean of each dependent variable was submitted to a 3-way RM ANOVA to test the main effects of and interaction between limb (dominant, non-dominant), load (20, 25, 30, and 35kg), and landing type (NL and FL). Significant interactions were submitted to simple main effects analysis and a Bonferroni correction was used for post-hoc comparisons to reduce the probability of committing type I error. Effect size was calculated for all significant main effects and interactions using partial omega squared (ω^2^) and pairwise comparisons using Cohen’s d (d) [38,39]. Alpha level was set *a priori* at P < 0.05. All statistical analysis were performed using SPSS (v23, IBM Corporation, Armonk, New York, USA). All dependent variables were checked for normal distribution using the Shapiro-Wilk test. Only knee abduction angle was not normally distributed and underwent a log10 transformation to achieve normality (p > 0.05). Since the data transformation did not affect the outcome of the RM ANOVAs, the untransformed data are presented below to facilitate interpretation of results.

## Results

Significant interactions and main effects for sex, limb, load and landing are presented below. Because the interactions between and main effects of load and landing are redundant between ANOVAs, only the findings for load and landing type from the analysis for purpose 1 are presented below.

### Effect of sex

The ANOVA revealed a significant 3-way interaction for peak knee abduction moment (*p* = 0.02, ω^2^ = 0.05). Post hoc analysis revealed that females exhibited greater peak knee abduction moment compared to males when landing with the 20 (*p* = 0.01, d = 0.42), 25 (*p* = 0.01, d = 0.42), and 35 kg loads (*p* < 0.001, d = 0.55) during the NL and with the 25 kg (*p* = 0.01, d = 0.42) and 35 kg (*p* < 0.01, d = 0.41) loads during the FL. Females exhibited greater peak knee abduction moment during the NL compared to FL with the 20 (*p* < 0.001, d = 0.38), 25 (*p* = 0.03, d = 0.20) and 35 kg loads (*p* < 0.001, d = 0.39), whereas, males only exhibited greater peak knee abduction moment during the NL with the 25 (*p* = 0.01, d = 0.22) and 30 kg loads (*p* = 0.04, d = 0.19). Neither sex exhibited a significant increase in peak knee abduction moment with each incremental addition of load during either the NL or FL.

**Fig 2:**
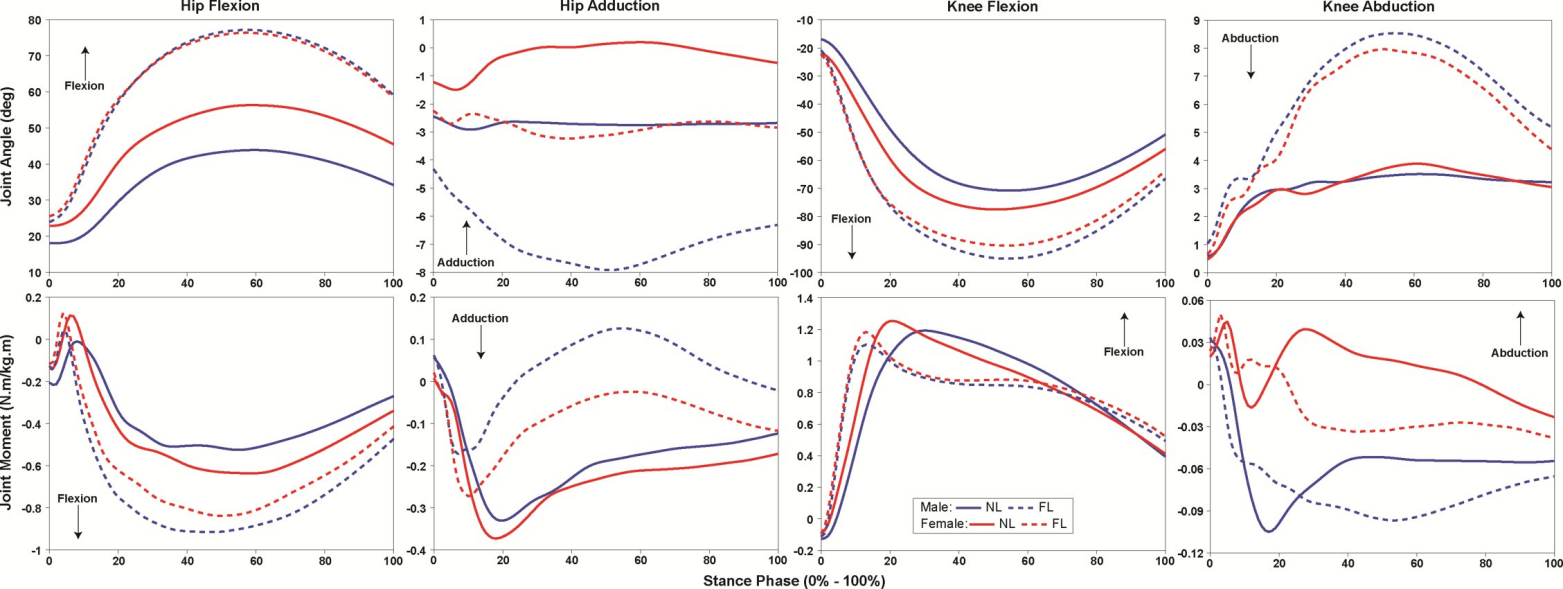
Landing phase (0–100%) hip and knee joint range of motion (ROM) and moments exhibited by males (blue) and females (red) during normal (NL, solid line) and flexed (FL, dashed line) landing.

There was a significant 2-way sex versus landing type interaction for peak hip flexion moment (*p* = 0.01, ω^2^ = 0.17). Both sexes increased hip flexion moment during FL compared to NL (*p* < 0.001, d = 1.35), but females exhibited greater peak hip flexion moment during the FL (*p* = 0.04, d = 0.59) and not during the NL (*p* = 0.97) compared to males (Fig 2).

There was a significant 2-way sex versus landing type interaction for hip (*p* = 0.01, ω^2^ = 0.16) and knee (*p* = 0.01, ω^2^ = 0.15) flexion ROM. Both sexes increased hip and knee flexion ROM during the FL compared to NL (*p* < 0.001, d = 14.48; *p* < 0.001, d = 12.47). Females, however, exhibited greater hip flexion ROM compared to males during the NL (*p* = 0.02, d = 4.67), but not during the FL (*p* = 0.44).

There was a significant 2-way sex versus load interaction for knee flexion ROM (*p* = 0.03, ω^2^ = 0.16). Males increased knee flexion ROM with the 25 compared to the 20 kg load (*p* = 0.01, d = 4.12), while females exhibited no significant change in knee flexion ROM between any load (*p* < 0.05).

**Fig 3:**
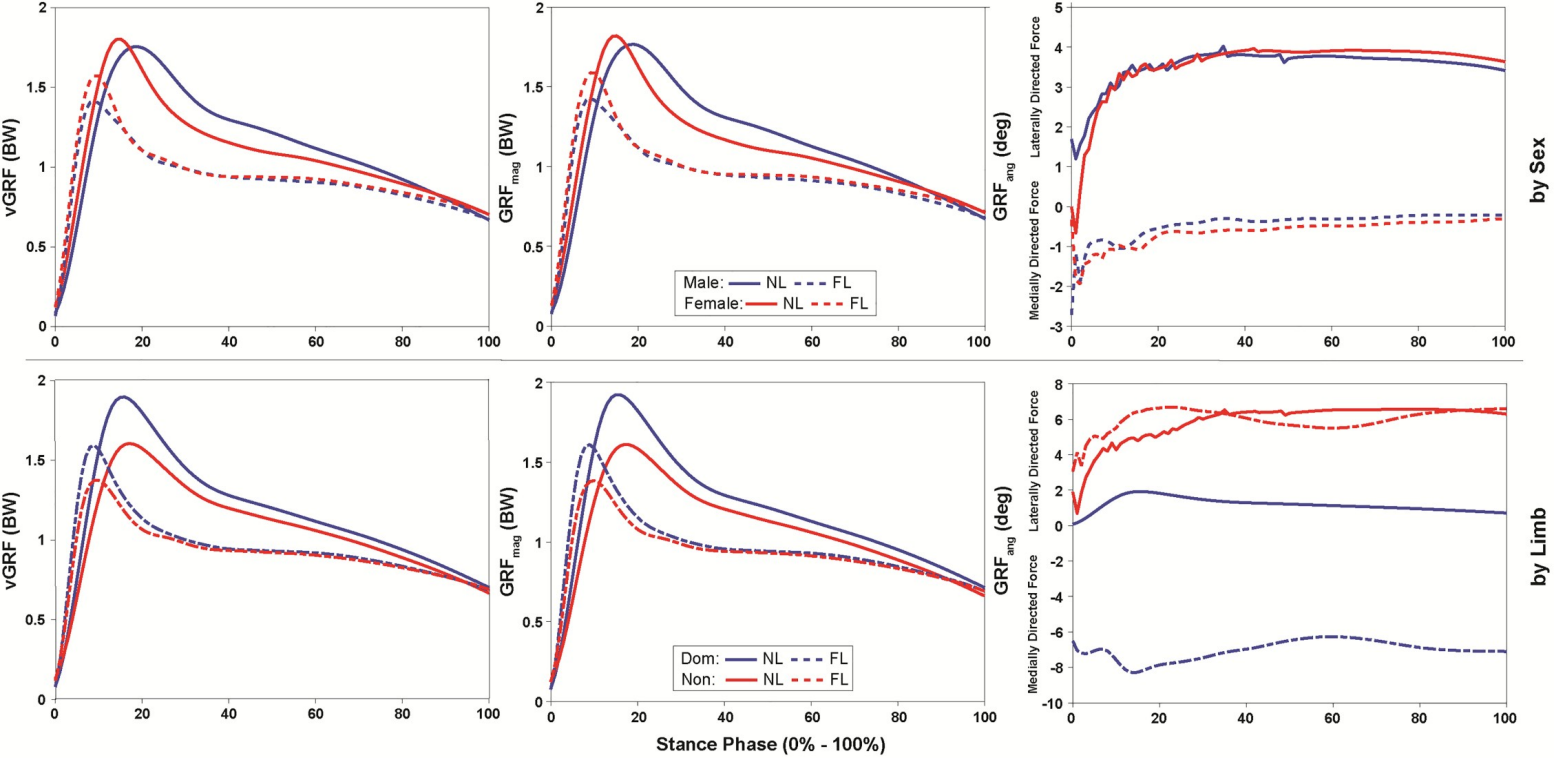
Landing phase (0–100%) vertical ground reaction force (vGRF) and frontal plane loading exhibited by males (blue) and females (red), and the dominant (blue) and non-dominant limb (red) during normal (NL, solid line) and flexed (FL, dashed line) landing.

There was a significant 2-way sex versus landing type interaction for vGRF (*p* = 0.01, ω^2^ = 0.17) and GRF_mag_ (*p* = 0.01, ω^2^ = 0.20). Both sexes decreased peak vGRF and GRF_mag_ during the FL compared to NL (*p* < 0.001, d = 1.68; *p* < 0.001, d = 1.55). Females, however, exhibited greater peak vGRF and GRF_mag_ compared to males during the FL (*p* < 0.01, d = 0.80; *p* = 0.01, d = 0.79), but not during the NL (*p* = 0.95 and *p* = 0.84) (Fig 3).

A main effect of sex was observed for hip and knee biomechanics. Specifically, females exhibited greater hip adduction (*p* < 0.001, ω^2^ = 0.26) ROM, and knee abduction (*p* = 0.01, ω^2^ = 0.20) moment compared to males.

### Effect of limb

There was a significant 3-way interaction for knee abduction ROM (*p* < 0.001, ω^2^ = 0.51). For the dominant limb, knee abduction ROM decreased during FL compared to NL with the 20 (*p* = 0.02, d = 0.88) and 25 kg loads (*p* = 0.03, d = 0.80); whereas, the non-dominant limb knee abduction ROM decreased during FL compared to NL with each load condition (*p* < 0.001, d > 0.99). Further, the non-dominant limb exhibited greater knee abduction ROM compared to dominant limb during NL with the 25 (*p* = 0.02, d = 1.71) and 30 kg loads (*p* < 0.001, d = 3.75), and during FL with the 25 kg load (*p* = 0.03, d = 1.52). For the non-dominant limb, knee abduction ROM was greater with 30 kg compared to the 20 (*p* < 0.001, d = 3.58), 25 (*p* = 0.03, d = 2.42), and 35 kg loads (*p* < 0.001, d = 3.36) during NL, but not during FL (*p* > 0.05).

The ANOVA revealed a significant 3-way interaction for knee flexion ROM (*p* < 0.001, ω^2^ = 0.70). Both limbs increased knee flexion ROM during FL compared to NL (*p* < 0.001, d = 12.41). During NL, the dominant limb exhibited great knee flexion ROM than the non-dominant limb with each load (*p* < 0.01, d = 4.90). During the FL, the dominant limb exhibited greater knee flexion ROM with the 20 (*p* = 0.02, d = 2.95), 30 (*p* = 0.03, d = 3.43) and 35 kg loads (*p* < 0.01, d = 5.13), whereas, the non-dominant limb exhibited greater knee flexion ROM with the 25 kg load (*p* < 0.001, d = 8.02). Further, during FL, the non-dominant limb exhibited greater knee flexion ROM with the 25 kg load compared to the 20 (*p* < 0.001, d = 11.17), 30 (*p* < 0.001, d = 9.97), and 35 kg loads (*p* < 0.001, d = 11.20), but similar differences in knee flexion ROM were not evident between load conditions for the dominant limb.

**Fig 4:**
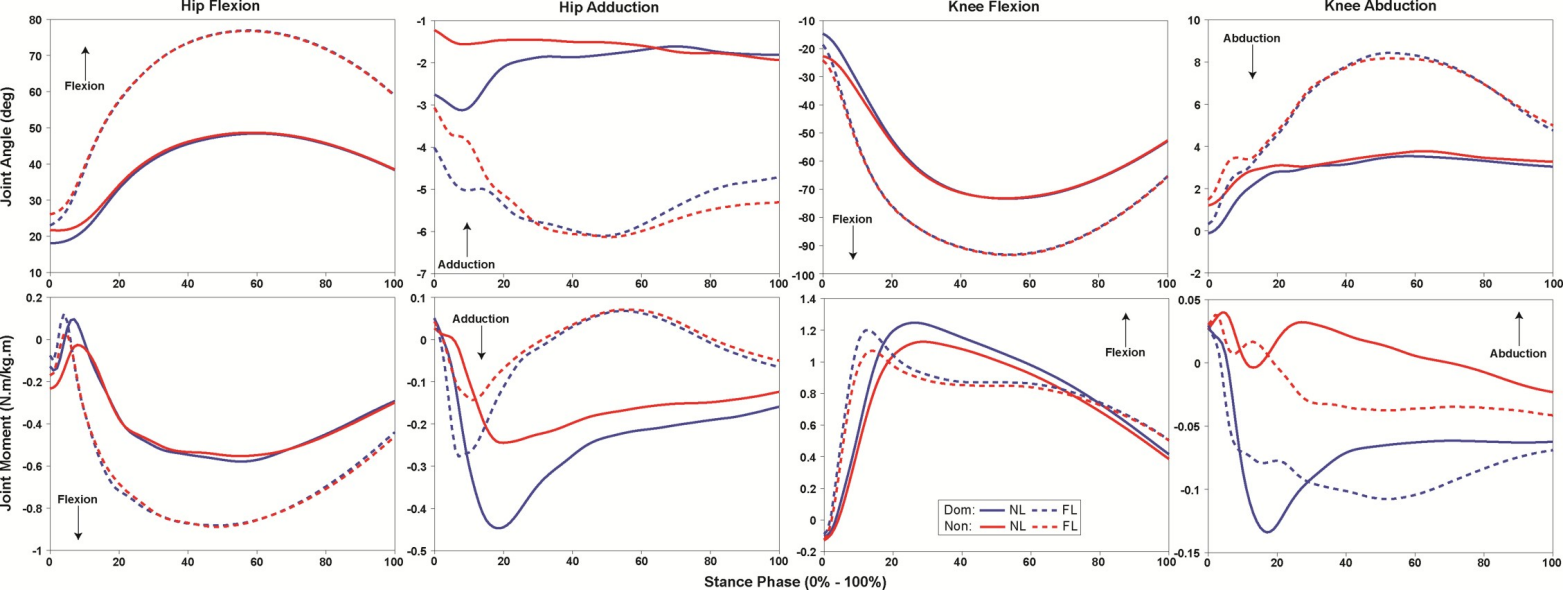
Landing phase (0–100%) hip and knee joint range of motion (ROM) and moments exhibited by the dominant (blue) and non-dominant limb (red) during normal (NL, solid line) and flexed (FL, dashed line) landing.

A significant 2-way limb versus landing type interaction was observed for hip adduction ROM (*p* = 0.04, ω^2^ = 0.40). Both limbs decreased hip adduction ROM during FL compared to NL (*p* = 0.02, d = 1.75). However, the dominant limb exhibited greater hip adduction ROM compared to the non-dominant limb during NL (*p* = 0.05, d = 1.42), but not FL (*p* = 0.37) (Fig 4).

A significant 2-way limb versus landing type interaction was observed for peak knee abduction moment (*p* = 0.01, ω^2^ = 0.14). The non-dominant limb exhibited greater knee abduction moment compared to the dominant limb during both the NL and FL (*p* < 0.001, d = 0.58; *p* < 0.001, d = 0.46) (Fig 4). But, the non-dominant limb decreased peak knee abduction moment during the FL compared to the NL (*p* < 0.001, d = 0.36), while dominant limb exhibited no significant difference between landings (*p* = 0.26).

The ANOVA revealed a significant 2-way limb versus landing type interaction for peak vGRF (*p* < 0.001, ω^2^ = 0.30) and GRF_mag_ (*p* < 0.01, ω^2^ = 0.23). During the FL, participants decreased vGRF and GRF_mag_ with both limbs (*p* < 0.001, d = 1.04; *p* < 0.001, d = 1.06), but peak vGRF and GRF_mag_ were greater for the dominant compared to non-dominant limb during both landings (NL: *p* < 0.001, d = 1.05 and *p* < 0.001, d = 1.11; FL: *p* = 0.03, d = 0.59 and *p* = 0.02, d = 0.61, respectively) (Fig 3).

The dominant limb exhibited greater peak vGRF (*p* < 0.01, ω^2^ = 0.23) and GRF_mag_ (*p* < 0.01, ω^2^ = 0.26), and a more medially-directed GRF_ang_ (*p* < 0.001, ω^2^ = 0.31) (Fig 3). Additionally, the dominant limb exhibited significantly smaller peak hip adduction moment (*p* = 0.01, ω^2^ = 0.19), and knee abduction (*p* < 0.001, ω^2^ = 0.41) moment and ROM (*p* < 0.01, ω^2^ = 0.26), but greater hip flexion ROM (*p* < 0.01, ω^2^ = 0.24), and knee flexion (*p* = 0.01, ω^2^ = 0.19) moment and ROM (*p* = 0.01, ω^2^ = 0.15) compared to the non-dominant limb (Fig 4).

### Effect of load

There was a significant 2-way load versus landing type interaction for knee flexion and abduction ROM (*p* < 0.001, ω^2^ = 0.24; *p* < 0.001, ω^2^ = 0.17). Participants increased knee flexion and abduction ROM during the FL compared to the NL with all loads (*p* < 0.001, d = 12.17; *p* = 0.01, d = 1.40) (Table 2). Further, knee flexion ROM was greater with the 25 kg compared to the 20 (*p* < 0.001, d = 6.62), 30 (*p* < 0.01, d = 6.17), and 35 kg loads (*p* < 0.001, d = 7.46) during FL, but similar differences were not evident during the NL (*p* > 0.05). Participants exhibited greater knee abduction ROM during NL with the 30 kg compared to the 20 (*p* = 0.01, d = 2.08) and 35 kg loads (*p* < 0.01, d = 2.14), with no differences observed during FL (*p* > 0.05) (Table 2).

**Table 2:** Mean (SD) joint range of motion (°) during normal (NL) and flexed (FL) drop landings.

	20 kg		25 kg		30 kg		35 kg	
	NL	FL	NL	FL	NL	FL	NL	FL
**Hip Flexion**	28.8 (11.6)‡	51.9 (7.3)‡	27.9 (10.6)‡	53.8 (9.3)‡	30.0 (12.1)‡	52.7 (8.4)‡	30.4 (12.2)‡	51.4 (9.2)‡
**Hip Adduction**	2.6 (2.5)‡	2.1 (2.4)‡	3.1 (2.8)‡	2.6 (2.7)‡	3.0 (3.0)‡	2.4 (3.1)‡	2.9 (2.8)‡	2.2 (2.8)‡
**Knee Flexion**	55.3 (11.5)‡	72.6 (9.5)#‡	53.5 (12.9)§‡	81.8 (14.5)*†§‡	57.2 (13.4)‡	73.1 (13.5)#‡	56.1 (14.0)#‡	72.0 (12.8)#‡
**Knee Abduction**	1.3 (1.9)†‡	1.0 (1.4)†‡	1.5 (2.1)‡	1.1 (1.8)‡	2.6 (2.2)*§‡	1.2 (1.7)*‡	1.2 (1.6)†‡	0.9 (1.4)‡

* Significant difference (p < 0.05) from 20 kg

# Significant difference (p < 0.05) from 25 kg

† Significant difference (p < 0.05) from 30 kg

§ Significant difference (p < 0.05) from 35 kg

‡ Significant difference (p < 0.05) between landing type

A significant 2-way load versus landing type interaction was observed for GRF_ang_ (*p* = 0.01, ω^2^ = 0.08). The GRF_ang_ was more medially-directed during the FL compared to NL with the 25 (*p* = 0.01, d = 1.16), 30 (*p* < 0.001, d = 2.28) and 35 kg (*p* < 0.001, d = 1.78) loads, but not with the 20 kg (*p* = 0.22) load (Table 3).

**Table 3:** Mean (SD) peak vGRF (BW), GRF_mag_ (BW) and GRF_ang_ (°) during normal (NL) and flexed (FL) drop landings.

	20 kg		25 kg		30 kg		35 kg	
	NL	FL	NL	FL	NL	FL	NL	FL
**Peak vGRF**	2.1 (0.3)#†§	1.7 (0.2)#†§	2.2 (0.3)*§	1.7 (0.2)*§	2.2 (0.3)*§	1.8 (0.2)*§	2.2 (0.3)*#†	1.9 (0.3)*#†
**GRF**_**mag**_	2.1 (0.3)#†§	1.7 (0.2)#†§	2.2 (0.3)*§	1.8 (0.2)*§	2.2 (0.3)*§	1.8 (0.2)*§	2.3 (0.3)*#†	1.9 (0.3)*#†
**GRF**_**ang**_	-6.2 (2.0)	-6.4 (1.8)	-6.2 (1.8)‡	-6.7 (1.7)‡	-5.7 (1.8)‡	-6.7 (1.7)‡	-5.7 (2.8)‡	-6.4 (2.9)‡

* Significant difference (p < 0.05) from 20 kg

# Significant difference (p < 0.05) from 25 kg

† Significant difference (p < 0.05) from 30 kg

§ Significant difference (p < 0.05) from 35 kg

‡ Significant difference (p < 0.05) between landing type

vGRF = vertical ground reaction force, GRF_mag_ = ground reaction force magnitude, GRF_ang_ = ground reaction force angle

A main effect of body borne load was observed for peak vGRF (*p* < 0.001, ω^2^ = 0.52) and GRF_mag_ (*p* < 0.001, ω^2^ = 0.50), knee flexion and abduction ROM (*p* = 0.02, ω^2^ = 0.19; *p* = 0.03, ω^2^ = 0.16), and peak hip (*p* < 0.001, ω^2^ = 0.37) and knee (*p* < 0.01, ω^2^ = 0.25) flexion moments (Tables 2–4). There was no main effect of body borne load on hip ROM during landing.

**Table 4:** Mean (SD) peak joint moments (N·m/kg·m) during normal (NL) and flexed (FL) drop landings.

	20 kg		25 kg		30 kg		35 kg	
	NL	FL	NL	FL	NL	FL	NL	FL
**Hip Flexion** (-)	-0.7 (0.2)†§	-1.0 (0.2)†§	-0.7 (0.2)†	-1.0 (0.2)†	-0.8 (0.3)*#	-1.1 (0.2)*#	-0.8 (0.3)*	-1.1 (0.3)*
**Hip Adduction** (-)	0.1 (0.1)	0.2 (0.1)	0.1 (0.1)	0.2 (0.1)	0.1 (0.1)	0.2 (0.1)	0.1 (0.1)	0.2 (0.2)
**Knee Flexion** (+)	1.4 (0.2)§	1.2 (0.2)§	1.5 (0.2)	1.3 (0.2)	1.4 (0.2)	1.3 (0.2)	0.4 (0.3)*	1.4 (0.2)*
**Knee Abduction** (+)	0.1 (0.1)	0.1 (0.1)	0.1 (0.1)	0.1 (0.1)	0.1 (0.1)	0.1 (0.1)	0.1 (0.1)	0.1 (0.0)

* Significant difference (p < 0.05) from 20 kg

# Significant difference (p < 0.05) from 25 kg

† Significant difference (p < 0.05) from 30 kg

§ Significant difference (p < 0.05) from 35 kg

### Effect of landing

During the FL, participants exhibited less peak vGRF (*p* < 0.001, ω^2^ = 0.75) and GRF_mag_ (*p* < 0.001, ω^2^ = 0.72), and a more medially-directed GRF_ang_ (*p* < 0.001, ω^2^ = 0.42) compared to NL (Fig 3). Further, participants exhibited greater peak hip flexion moment (*p* < 0.001, ω^2^ = 0.72) and ROM (*p* < 0.001, ω^2^ = 0.82) and knee flexion (*p* < 0.001, ω^2^ = 0.78) ROM, but significantly less hip adduction and knee abduction ROM (*p* < 0.001, ω^2^ = 0.37; *p* < 0.001, ω^2^ = 0.52) and moments (*p* < 0.001, ω^2^ = 0.38; *p* < 0.001, ω^2^ = 0.31), and knee flexion moment (*p* < 0.001, ω^2^ = 0.57) during the FL (Figs 2 and 4).

## Discussion

Landing with body borne load may increase risk of musculoskeletal injury [11,12]. In agreement with previous research, participants exhibited a significant 5 to 10% increase in peak vGRF when landing with each addition of body borne load [11]. Dissipating these large landing forces can strain musculature surrounding a joint, particularly at the knee [8]. The current participants exhibited a significant increase in hip and knee flexion joint moments with the addition of body borne load. Larger knee flexion moments are reported to be an indicator of knee soft-tissue loading [40], particularly with an extended limb. Participants were only able to achieve a greater knee flexion range of motion during landing with one of the lighter (25 kg) body borne loads, and exhibited no significant change in hip flexion range of motion with load. When landing with heavy body borne loads, an extended lower limb posture may prevent collapse of the lower limb [41], but may also result in greater transmission of the GRFs to the musculoskeletal system, increasing risk of injury [42]. Future work is warranted to address how adaptations of other lower limb biomechanics, at the hip and ankle, impact knee biomechanics and risk of injury at the joint during dynamic tasks, particularly with the addition of heavy body borne loads common to military training.

This study demonstrated that using greater lower limb flexion when landing with body borne load is, indeed, attainable and may reduce risk of musculoskeletal injury [11,29]. Specifically, when instructed, participants increased hip and knee flexion range of motion by 22° and 19° during landing. In agreement with previous research [30,31], increased lower limb flexion during landing reduced peak vertical GRF by 17%. This reduction in peak vGRF may result from greater absorption of the landing forces by the lower limb musculature that occurs when using greater flexion during landing [11] and coincide with decreased injury risk. Additionally, during the flexed landings, the magnitude of the frontal plane GRF vector was significantly smaller and more medially-directed. The medially-directed GRF vector may aid participants in the reduction of frontal plane hip and knee biomechanics related to knee injury and loading of the joint’s soft-tissue when donning heavy body borne loads (25–35 kg) during landing. These results, however, may be interpreted with caution, as the observed effect of the load and landing type interaction for GRF_ang_ was trivial. Regardless, the current participants exhibited a significant decrease in hip adduction and knee abduction range of motion and joint moments during the flexed landings. These biomechanical patterns may reduce dynamic knee valgus loads and decrease strain on the ACL [30] during flexed landings. Thus, instructing military personnel to increase lower limb flexion during landing may be a beneficial strategy to reduce the incidence of musculoskeletal injury, particularly at the knee, during training.

Females are more prone to musculoskeletal injury than their male counterparts [4,27,28]. Females’ injury risk may be attributed to the large vertical GRFs [24] and asymmetries of frontal plane hip and knee biomechanics [21,25,26] they exhibit during landing. The current outcomes suggest that landing with heavy body borne load may further increase female risk of musculoskeletal injury. During the loaded landings, females exhibited hip and knee biomechanics related to knee valgus loading and elevated risk of injury. Notably, females exhibited 1.7° greater hip adduction range of motion and 0.06 N.m/kg.m greater peak knee abduction moment compared to males when landing with body borne load, regardless of landing type. Independent of neuromuscular control and normalized to height and weight, females demonstrate increased knee abduction moment than males during a drop landing [43]. These large knee abduction moments are a prospective predictor of ACL injury [5,8] and when coupled with excessive hip adduction may further increase strain on the ACL, contributing to females’ high rate of injury [8,9,20].

For the current study, both sexes exhibited greater hip and knee flexion during the flexed landings. Females, however, did not exhibit a similar reduction in landing forces and subsequent injury risk as their male counterparts. Females decreased peak vGRF 12% during flexed landings, but still exhibited 0.2 BWs larger vGRF than males. To control these large landing GRFs, females used greater hip flexion moment compared to males. This strategy is a noted sex dimorphism for females during landing [21–23], and has been attributed to poor neuromuscular control at the hip [8,20,44] and disproportionate strength of the hip abductor and knee flexor muscles [8,44]. Neuromuscular training has been shown to reduce females’ knee injury risk [16,44] by focusing on increasing hip abduction strength [45]. But, it is unknown if similar training interventions can improve females’ lower limb biomechanics and successfully mitigate their injury risk during activity with heavy body borne load.

While the existing literature is inconclusive on limb differences during landing [23,24], with the addition of body borne load participants may rely more on the stronger dominant limb [46,47] to safely attenuate the impact forces for both normal and flexed landings [16]. Participants’ dominant limb exhibited greater peak vertical (NL: 12%, FL: 8%) and frontal (NL: 13%, FL: 7%) GRFs and peak knee flexion moment than the non-dominant limb. Whereas, the weaker non-dominant limb may lack the strength and/or neuromuscular control to prevent excessive out of plane knee biomechanics implicated in injury [8,17] during loaded landings. Contrary findings have reported the non-dominant limb exhibiting greater gluteus medius muscle force at peak vertical GRF than the dominant limb, which would suggest greater hip abductor strength and less knee valgus motion [48]. Currently, the non-dominant limb exhibited a more laterally-directed frontal plane GRF vector, which may act to push the knee into dynamic valgus [8,18] producing the greater knee abduction joint moments currently observed during landing. Further, the non-dominant limb’s reported decrement in strength and neuromuscular control [8,17] may limit the limb’s ability to produce similar reductions in knee abduction range of motion and moment as the dominant limb during the flexed landings, subsequently increasing its injury risk. Future work is warranted to determine how discrepancies in strength and neuromuscular control between limbs elevates musculoskeletal injury risk during military training-related tasks.

### Limitations

This study may be limited by the landing task and participants. Although, military personnel may land from heights greater than 30 cm during training, previous experimental outcomes demonstrate landing from 22 and 27 cm reportedly alters lower limb biomechanics, in particular knee joint kinematics and kinetics [23,31], and may be suitable to assess knee injury risk between sexes and limbs. However, landing from heights greater than 30 cm does occur during military-activities and warrants further study as it may illicit larger changes in lower limb biomechanics during landing. While using greater lower limb flexion when landing with heavy body borne load was attainable by the current participants in a controlled laboratory environment, this may not translate to the unconstrained environment of military training. For instance, Pappas and Carpes [25] reported athletes exhibited greater hip adduction and knee abduction angles during a forward landing task, in which participants jumped forward then landed, compared to an isolated drop landing task. Further research is necessary to determine if a reduction in injury risk is achievable for personnel during unconstrained military training-based tasks. The chosen participants self-reported the ability to carry 75 pounds, but were not required to have military load carriage experience. Participants with load carriage experience may exhibit strength differences that alter landing biomechanics compared to participants unaccustomed to donning body borne load, yet we are unaware of previous research that has compared landing biomechanics between experienced and inexperienced load carriers.

## Conclusions

Landing with body borne load increased participants’ risk of musculoskeletal injury. Specifically, participants exhibited greater GRFs and hip and knee joint moments with the addition of body borne load during landing. Increasing lower limb flexion during landing may decrease this risk of musculoskeletal injury, as participants were able to decrease GRFs and hip and knee biomechanics related to knee injury during the flexed landings. Both females and the non-dominant limb may be at greater risk of suffering a musculoskeletal injury during landing. Females exhibited larger GRFs, increased hip adduction range of motion, and greater knee abduction moments compared to males. Whereas, the non-dominant limb increased knee abduction moments and exhibited a more laterally-directed frontal plane GRF vector compared to the dominant limb during the loaded landings. Yet, increasing lower limb flexion landings does not appear to produce similar reductions in lower limb biomechanics related to injury risk for both females and the non-dominant limb during landing.

## Supporting information

S1 Table Peak vGRF (BW), GRFmag (BW) and GRFang (°) between normal (NL) and flexed (FL) drop landings. vGRF = vertical ground reaction force, GRFmag = ground reaction force magnitude, GRFang = ground reaction force angle
(PDF)

S2 Table Joint range of motion (°) between normal (NL) and flexed (FL) drop landings. (PDF)

S3 Table Peak joint moments (N·m/kg·m) between normal (NL) and flexed (FL) drop landings. (PDF)

S4 Table Peak vGRF (BW), GRFmag (BW) and GRFang (°) between sexes during normal (NL) and flexed (FL) drop landings. vGRF = vertical ground reaction force, GRFmag = ground reaction force magnitude, GRFang = ground reaction force angle
(PDF)

S5 Table Joint range of motion (°) between sexes during normal (NL) and flexed (FL) drop landings. (PDF)

S6 Table Peak joint moments (N·m/kg·m) between sexes during normal (NL) and flexed (FL) drop landings. (PDF)

S7 Table Peak vGRF (BW), GRFmag (BW) and GRFang (°) between limbs during normal (NL) and flexed (FL) drop landings. vGRF = vertical ground reaction force, GRFmag = ground reaction force magnitude, GRFang = ground reaction force angle
(PDF)

S8 Table Joint range of motion (°) between limbs during normal (NL) and flexed (FL) drop landings. (PDF)

S9 Table Peak joint moments (N·m/kg·m) between limbs during normal (NL) and flexed (FL) drop landings. (PDF)
