# Supplementary material for: Sex and limb impact biomechanics associated with risk of injury during drop landing with body borne load
Source: PLoS One. 2019 Feb 6;14(2):e0211129. doi: 10.1371/journal.pone.0211129 (PMC6364912; doi:10.1371/journal.pone.0211129)
Supplement: S1 Table — vGRF = vertical ground reaction force, GRFmag = ground reaction force magnitude, GRFang = ground reaction force angle (PDF) [file pone.0211129.s001.pdf]

S1 Table: Peak vGRF (BW), GRF_{mag} (BW) and GRF_{ang} (°) between normal (NL) and flexed (FL) drop landings.

		Mean	Min	Max	95% Confidence Interval	***p* – value Main Effect (Land)**
**Peak vGRF**	**NL**	2.16	1.41	3.04	2.07 – 2.24	< 0.001
	**FL**	1.81	1.28	2.48	1.75 – 1.87	
**GRF_{mag}**	**NL**	2.16	1.38	3.07	2.08 – 2.25	< 0.001
	**FL**	1.84	1.28	2.52	1.78 – 1.91	
**GRF_{ang}**	**NL**	-6.09	-10.27	1.83	-6.63 – -5.54	< 0.001
	**FL**	-6.70	-11.03	0.73	-7.23 – -6.17	

vGRF = vertical ground reaction force, GRF_{mag} = ground reaction force magnitude, GRF_{ang} = ground reaction force angle
