# Supplementary material for: Sex and limb impact biomechanics associated with risk of injury during drop landing with body borne load
Source: PLoS One. 2019 Feb 6;14(2):e0211129. doi: 10.1371/journal.pone.0211129 (PMC6364912; doi:10.1371/journal.pone.0211129)
Supplement: S2 Table — (PDF) [file pone.0211129.s002.pdf]

S2 Table: Joint range of motion (°) between normal (NL) and flexed (FL) drop landings.

		Mean	Min	Max	95% Confidence Interval	*p* - value Main Effect (Land)
**Hip Flexion**	**NL**	30.36	7.03	50.11	27.23 – 33.48	< 0.001
	**FL**	52.18	31.33	66.32	49.66 – 54.69	
**Hip Adduction**	**NL**	3.14	0.00	11.58	2.63 – 3.66	< 0.001
	**FL**	2.44	0.00	10.90	1.92 – 2.95	
**Knee Flexion**	**NL**	55.88	18.14	76.01	52.65 – 59.11	< 0.001
	**FL**	73.92	33.20	96.58	70.57 – 77.26	
**Knee Abduction**	**NL**	1.69	0.05	7.50	1.34 – 2.03	< 0.001
	**FL**	1.09	0.00	6.02	0.78 – 1.41	
