# Supplementary material for: Sex and limb impact biomechanics associated with risk of injury during drop landing with body borne load
Source: PLoS One. 2019 Feb 6;14(2):e0211129. doi: 10.1371/journal.pone.0211129 (PMC6364912; doi:10.1371/journal.pone.0211129)
Supplement: S3 Table — (PDF) [file pone.0211129.s003.pdf]

S3 Table: Peak joint moments (N·m/kg·m) between normal (NL) and flexed (FL) drop landings.

		Mean	Min	Max	95% Confidence Interval	*p* - value Main Effect (Land)
**Hip Flexion (-)**	**NL**	-0.77	-2.13	-0.21	-0.86 – -0.69	< 0.001
	**FL**	-1.02	-2.12	-0.62	-1.09 – -0.95	
**Hip Adduction (-)**	**NL**	0.09	-0.12	0.35	0.06 – 0.12	< 0.001
	**FL**	0.16	-0.04	0.71	0.12 – 0.21	
**Knee Flexion (+)**	**NL**	1.45	0.92	1.99	1.38 – 1.51	< 0.001
	**FL**	1.32	0.89	1.83	1.26 – 1.38	
**Knee Abduction (+)**	**NL**	0.13	0.01	0.37	0.11 – 0.15	< 0.001
	**FL**	0.11	0.01	0.31	0.10 – 0.13	
