# Supplementary material for: Sex and limb impact biomechanics associated with risk of injury during drop landing with body borne load
Source: PLoS One. 2019 Feb 6;14(2):e0211129. doi: 10.1371/journal.pone.0211129 (PMC6364912; doi:10.1371/journal.pone.0211129)
Supplement: S4 Table — vGRF = vertical ground reaction force, GRFmag = ground reaction force magnitude, GRFang = ground reaction force angle (PDF) [file pone.0211129.s004.pdf]

S4 Table: Peak vGRF (BW), GRF_{mag} (BW) and GRF_{ang} (°) between sexes during normal (NL) and flexed (FL) drop landings.

		Female				Male				*p* - value	
					95% Confidence				95% Confidence	Main Effect	
		**Mean**	**Min**	**Max**	**Interval**	**Mean**	**Min**	**Max**	**Interval**	**Sex**	**Land**
**Peak vGRF**	**NL**	2.15	1.55	2.91	2.02 – 2.29	2.16	1.48	2.89	2.06 – 2.26	0.17	< 0.001
	**FL**	1.90	1.34	2.48	1.81 – 2.00	1.71	1.34	2.25	1.64 – 1.79		
**GRF_{mag}**	**NL**	2.15	1.53	2.89	2.01 – 2.30	2.17	1.43	2.97	2.07 – 2.28	0.21	< 0.001
	**FL**	1.94	1.36	2.52	1.84 – 2.04	1.75	1.36	2.27	1.67 – 1.83		
**GRF_{ang}**	**NL**	-6.58	-10.02	-2.09	-7.45 – -5.71	-5.60	-9.07	1.05	-6.26 – -4.94	0.05	< 0.001
	**FL**	-7.24	-10.57	-3.31	-8.09 – -6.39	-6.16	-9.59	0.52	-6.81 – -5.52		

vGRF = vertical ground reaction force, GRF_{mag} = ground reaction force magnitude, GRF_{ang} = ground reaction force angle
