# Supplementary material for: Sex and limb impact biomechanics associated with risk of injury during drop landing with body borne load
Source: PLoS One. 2019 Feb 6;14(2):e0211129. doi: 10.1371/journal.pone.0211129 (PMC6364912; doi:10.1371/journal.pone.0211129)
Supplement: S5 Table — (PDF) [file pone.0211129.s005.pdf]

S5 Table: Joint range of motion (°) between sexes during normal (NL) and flexed (FL) drop landings.

		Female				Male				*p* - value	
		Mean	Min	Max	95% Confidence Interval	Mean	Min	Max	95% Confidence Interval	Main Effect Sex	Main Effect Land
**Hip Flexion**	**NL**	34.32	15.41	47.84	29.33 – 39.31	26.39	7.44	48.43	22.62 – 30.16	0.17	< 0.001
	**FL**	51.21	32.93	64.49	47.20 – 55.22	53.15	35.67	65.99	50.12 – 56.18		
**Hip Adduction**	**NL**	3.97	0.33	11.58	3.15 – 4.80	2.31	0.00	7.71	1.69 – 2.93	< 0.01	< 0.001
	**FL**	3.30	0.07	10.82	2.48 – 4.12	1.57	0.00	7.22	0.96 – 2.19		
**Knee Flexion**	**NL**	57.23	21.18	74.77	52.09 – 62.38	54.53	26.03	73.90	50.64 – 58.42	0.54	< 0.001
	**FL**	70.85	33.20	88.95	65.52 – 76.19	76.99	57.63	95.02	72.95 – 81.02		
**Knee Abduction**	**NL**	1.83	0.05	7.22	1.28 – 2.38	1.55	0.09	5.56	1.13 – 1.97	0.33	< 0.001
	**FL**	1.26	0.00	5.72	0.76 – 1.76	0.92	0.00	5.08	0.55 – 1.30		
