# Supplementary material for: Sex and limb impact biomechanics associated with risk of injury during drop landing with body borne load
Source: PLoS One. 2019 Feb 6;14(2):e0211129. doi: 10.1371/journal.pone.0211129 (PMC6364912; doi:10.1371/journal.pone.0211129)
Supplement: S6 Table — (PDF) [file pone.0211129.s006.pdf]

S6 Table: Peak joint moments (N·m/kg·m) between sexes during normal (NL) and flexed (FL) drop landings.

		Female				Male				*p* - value	
		**Mean**	**Min**	**Max**	**95% Confidence Interval**	**Mean**	**Min**	**Max**	**95% Confidence Interval**	**Main Effect Sex**	**Main Effect Land**
**Hip Flexion (-)**	**NL**	-0.77	-1.24	-0.49	-0.91 – -0.63	-0.77	-2.06	-0.21	-0.88 – -0.67	0.30	< 0.001
	**FL**	-0.94	-1.32	-0.65	-1.05 – -0.83	-1.09	-2.11	-0.71	-1.18 – -1.01		
**Hip Adduction (-)**	**NL**	0.07	-0.07	0.23	0.03 – 0.12	0.11	-0.09	0.35	0.08 – 0.14	0.08	< 0.001
	**FL**	0.12	-0.02	0.37	0.05 – 0.19	0.20	-0.02	0.70	0.15 – 0.26		
**Knee Flexion (+)**	**NL**	1.49	1.01	1.92	1.39 – 1.59	1.40	1.00	1.91	1.33 – 1.48	0.10	< 0.001
	**FL**	1.37	1.01	1.83	1.28 – 1.46	1.27	0.89	1.66	1.20 – 1.34		
**Knee Abduction (+)**	**NL**	0.15	0.04	0.34	0.13 – 0.18	0.10	0.01	0.29	0.08 – 0.12	0.01	< 0.001
	**FL**	0.13	0.03	0.31	0.11 – 0.16	0.09	0.02	0.24	0.08 – 0.11		
