# Supplementary material for: Sex and limb impact biomechanics associated with risk of injury during drop landing with body borne load
Source: PLoS One. 2019 Feb 6;14(2):e0211129. doi: 10.1371/journal.pone.0211129 (PMC6364912; doi:10.1371/journal.pone.0211129)
Supplement: S7 Table — vGRF = vertical ground reaction force, GRFmag = ground reaction force magnitude, GRFang = ground reaction force angle (PDF) [file pone.0211129.s007.pdf]

S7 Table: Peak vGRF (BW), GRF_{mag} (BW) and GRF_{ang} (°) between limbs during normal (NL) and flexed (FL) drop landings.

		Dominant				Non-Dominant				*p* - value	
					95% Confidence				95% Confidence	Main Effect	
		**Mean**	**Min**	**Max**	**Interval**	**Mean**	**Min**	**Max**	**Interval**	**Limb**	**Land**
**Peak vGRF**	**NL**	2.29	1.52	3.18	2.19 – 2.40	2.02	1.30	2.89	1.92 – 2.13	< 0.01	< 0.001
	**FL**	1.85	1.34	2.55	1.77 – 1.94	1.71	1.21	2.41	1.62 – 1.80		
**GRF_{mag}**	**NL**	2.31	1.54	3.18	2.20 – 2.42	2.02	1.22	2.95	1.91 – 2.13	< 0.01	< 0.001
	**FL**	1.89	1.35	2.59	1.80 – 1.98	1.75	1.22	2.46	1.66 – 1.84		
**GRF_{ang}**	**NL**	-6.59	-11.14	0.48	-7.27 – -5.91	-5.32	-9.39	3.19	-8.03 – -6.62	< 0.001	< 0.001
	**FL**	-7.32	-12.42	-0.04	-5.92 – -4.72	-5.78	-9.63	1.51	-6.40 – -5.17		

vGRF = vertical ground reaction force, GRF_{mag} = ground reaction force magnitude, GRF_{ang} = ground reaction force angle
