# Supplementary material for: Sex and limb impact biomechanics associated with risk of injury during drop landing with body borne load
Source: PLoS One. 2019 Feb 6;14(2):e0211129. doi: 10.1371/journal.pone.0211129 (PMC6364912; doi:10.1371/journal.pone.0211129)
Supplement: S8 Table — (PDF) [file pone.0211129.s008.pdf]

S8 Table: Joint range of motion (°) between limbs during normal (NL) and flexed (FL) drop landings.

		Dominant				Non-Dominant				*p* - value	
										Main Effect	
		Mean	Min	Max	95% Confidence Interval	Mean	Min	Max	95% Confidence Interval	Limb	Land
**Hip Flexion**	**NL**	30.88	8.60	51.92	27.46 – 34.30	27.67	5.47	48.30	24.29 – 31.05	< 0.01	< 0.001
	**FL**	53.95	32.65	68.46	51.32 – 56.58	50.93	30.01	64.17	48.40 – 53.47		
**Hip Adduction**	**NL**	3.45	0.00	11.09	2.69 – 4.21	2.37	0.00	12.06	1.58 – 3.17	0.13	< 0.001
	**FL**	2.45	0.00	9.70	1.76 – 3.13	1.96	0.00	12.09	1.09 – 2.83		
**Knee Flexion**	**NL**	59.44	28.54	79.16	55.98 – 62.89	51.59	7.74	72.86	47.69 – 55.49	0.01	< 0.001
	**FL**	75.28	31.94	96.25	71.68 – 78.89	74.44	34.46	96.91	70.64 – 78.24		
**Knee Abduction**	**NL**	1.09	0.00	7.11	0.71 – 1.47	2.21	0.09	7.90	1.76 – 2.66	< 0.01	< 0.001
	**FL**	0.86	0.00	5.69	0.56 – 1.15	1.24	0.00	6.34	0.80 – 1.68		
