# Supplementary material for: Sex and limb impact biomechanics associated with risk of injury during drop landing with body borne load
Source: PLoS One. 2019 Feb 6;14(2):e0211129. doi: 10.1371/journal.pone.0211129 (PMC6364912; doi:10.1371/journal.pone.0211129)
Supplement: S9 Table — (PDF) [file pone.0211129.s009.pdf]

S9 Table: Peak joint moments (N·m/kg·m) between limbs during normal (NL) and flexed (FL) drop landings.

		Dominant				Non-Dominant				*p* - value	
										Main Effect	
		Mean	Min	Max	95% Confidence Interval	Mean	Min	Max	95% Confidence Interval	Limb	Land
**Hip Flexion (-)**	**NL**	-0.77	-1.84	-0.09	-0.86 – -0.67	-0.78	-2.42	-0.33	-0.91 – -0.66	0.78	< 0.001
	**FL**	-1.03	-1.77	-0.59	-1.10 – -0.96	-1.05	-2.47	-0.66	-1.16 – -0.94		
**Hip Adduction (-)**	**NL**	0.07	-0.12	0.27	0.05 – 0.10	0.12	-0.12	0.43	0.09 – 0.15	0.01	< 0.001
	**FL**	0.15	-0.05	0.63	0.11 – 0.20	0.19	-0.04	0.79	0.14 – 0.24		
**Knee Flexion (+)**	**NL**	1.50	0.98	2.03	1.43 – 1.57	1.38	0.87	1.94	1.30 – 1.45	0.01	< 0.001
	**FL**	1.34	0.89	1.92	1.27 – 1.42	1.27	0.89	1.74	1.21 – 1.32		
**Knee Abduction (+)**	**NL**	0.09	-0.01	0.34	0.07 – 0.11	0.15	0.02	0.39	0.13 – 0.18	< 0.001	< 0.001
	**FL**	0.08	0.00	0.31	0.07 – 0.10	0.13	0.02	0.31	0.11 – 0.15		
